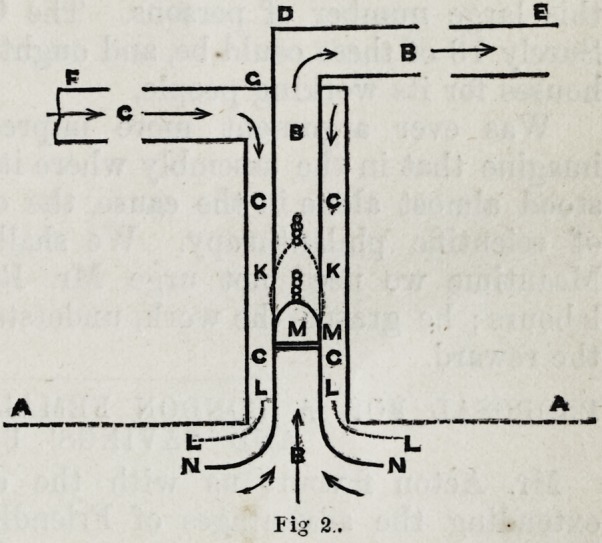# M'Kinnell's Ventilator

**Published:** 1857-10

**Authors:** 


### MISCELLANEA.

### M'KINNELL'S VENTILATOR.

We have, on repeated occasions, laid before the readers of this Review the particulars of the various plans invented by different authors for securing an efficient ventilation. At the same time we have generally opposed all modes of ventilation except the simple, natural system.

During the past quarter Mr. M'Kinnell, of Glasgow, has brought before us, in the way of direct experiment, his system of ventilation. And we are bound to record the entire success of his demonstration in so far as such demonstration was practicable on a small scale. The plan is described as follows in Professor Nicol's *Cyclopœdia of the Physical Sciences.*

"The method adverted to is founded on the circumstance that *one* tube, provided it be sufficiently capacious, may serve, at one and the same instant, for abduction and induction, the centre being occupied by a column of warm outgoing air, while, towards the circumference, a stream of cold air is rushing inwards. Although a partial knowledge of the fact of counter currents taking place in a single opening was possessed by the earlier writers on ventilation, Mr. M'Kinnell, of Glasgow, was the first, it is believed, to discover, and draw attention to this unvariable arrangement of aerial currents in the circumstances described. Openings of sufficient capacity, however, to admit of the unimpeded movements of these currents, would, in the climate of Britain, be intolerable. But on investigation, it was found that the same effects could be obtained in smaller space, by relieving the ascending and descending currents from mutual contact. Mr. M'Kinnell's patent ventilator is constructed on these principles. It consists mainly of two tubes arranged concentrically, the inner discharging the vitiated air, while the fresh supply flows down the outer tube. It is almost automatic in its action, requiring little or no attention in ordinary circumstances. It removes the air as it is vitiated, and supplies its place with pure air, in the exact amount required, in currents so gentle as scarcely to be perceptible. The contrivance also possesses this great advantage, that it can be introduced, and acts as effectively, between the ceiling and floor of the lower stories of buildings, as in apartments having immediate access."

The following diagrams illustrate the plan more strikingly.

Fig. 1.

Fig. 1. General view of the arrangement and particular combination of the tubes. B. and C. Two tubes arranged concentrically and opening into the interior of an apartment at the line of ceiling A. A. A. They are represented also as opening outwards, by the roof of the house, in a vertical direction. The inner tube *invariably* discharges the vitiated air, while the fresh supply of air *invariably* flows down the outer tube, as indicated by the arrows. Fig. 2. A horizontal or an oblique direction may be given to one or other or both of the concentric tubes, so that the apparatus can be introduced and acts as effectively, between the ceiling and floors of the lower stories of buildings, as in apartments which have access to the roof.

A portion of the inner tube, M. L. N., at the end towards the ceiling, is so adjusted that it slides up or down by a telescopic motion, so that when drawn up its trumpet-shaped end takes up the position indicated by the dotted lines K. L. L. (fig. 2) and so closes completely, or partially, the aperture which admits the fresh air. The trumpet-shaped form of the tube which conveys the out-going current, deflects the currents of fresh air which are passing down through the circumferential space of the larger tube C, so that the force of the descending current is broken, and

Fig. 2.

is made to spread out into the space to be ventilated; the trumpet expansion also serves to collect and guide the outgoing currents which pass up through the central tube. An ornamental moulding may be applied to this opening; and thus may make a beautiful centre-piece in the ceiling of a room. The double transverse lines between K and L indicate the position of a diaphragm.

Independently of all theoretical argument, we consider M'Kinnell's system as well worthy of extensive application. It is simple; it aims at approaching a natural law; and with its good properties it certainly blends no evil ones, which is saying an immense deal for a ventilator.

## Figures and Tables

**Fig. 1. f1:**
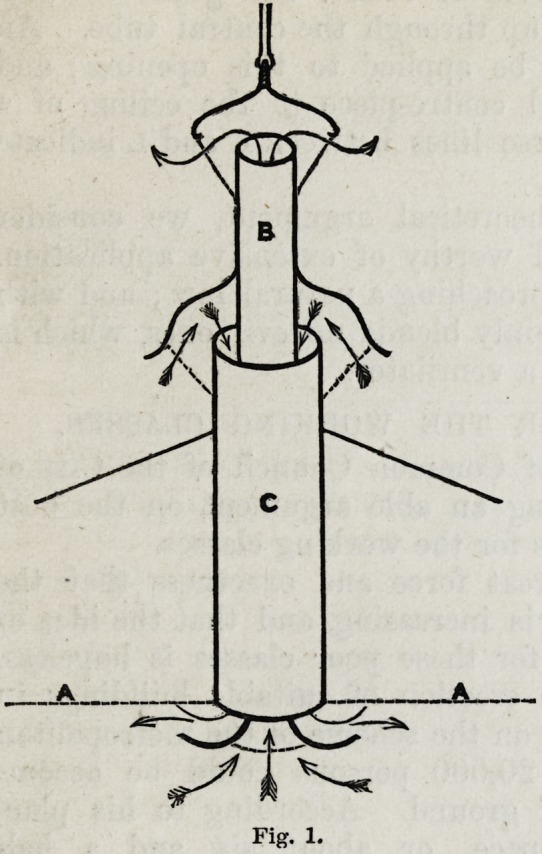


**Fig 2. f2:**